# Supplementary material for: Effects of Non-Ionic Micelles on the Acid-Base Equilibria of a Weak Polyelectrolyte
Source: Polymers (Basel). 2022 May 9;14(9):1926. doi: 10.3390/polym14091926 (PMC9100363; doi:10.3390/polym14091926)
Supplement: Supplementary file 1 [file polymers-14-01926-s001.zip › polymers-1708910-supplementary.pdf]

Supplementary information

Effects of non-ionic micelles on the acid-base equilibria of a weak polyelectrolyte

Evgenee Yekymov [1], David Attia [1], Yael Levi-Kalisman [2], Ronit Bitton[1,3] and Rachel Yerushalmi-Rozen [1,3,*]

[1] Department of Chemical Engineering, Ben-Gurion University of the Negev, 84105 Beer-Sheva, Israel.

[2] The Center for Nanoscience and Nanotechnology, and The Institute of Life Sciences at The Hebrew University of Jerusalem, Jerusalem 9190401, Israel.

[3] The Ilse Katz Institute for Nanoscience and Technology, Ben-Gurion University of the Negev, 84105 Beer-Sheva, Israel.

* Correspondence: rachely@bgu.ac.il

In this study we investigated the effect of self-assembling polymers and surfactants on the titration curves of a weak polyelectrolyte, polyacrylic acid (PAA). Supplementary data and detailed analysis complementing those presented in the main text are presented here.

Titration curves calculated via FTIR-ATR measurements

ATR-FTIR spectra of PAA are presented in Figure S1. The acid form (Figure S1 A) is characterized by the fingerprint pattern of C=O, CH_2 and C-O bands in 1717, 1455 and 1265 cm^{-}

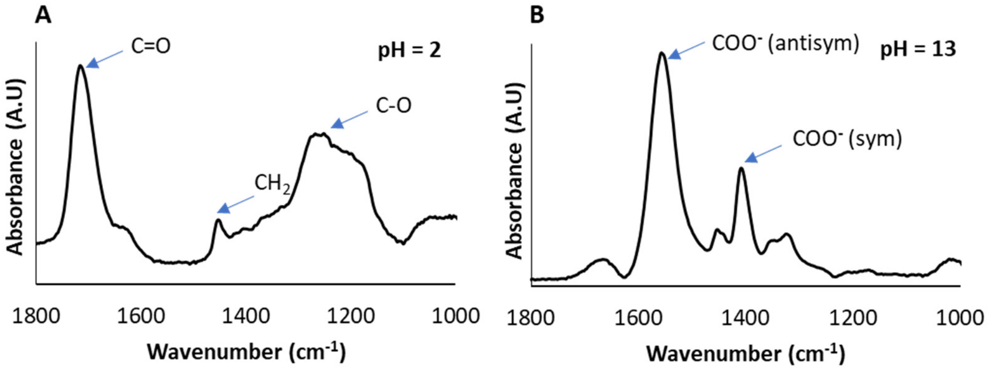

[1][1], respectively. The base form (Figure S1 B), with COO^- symmetric and antisymmetric bands at 1408 and 1562 cm^{-1}.

Figure S1. IR-Spectra of aqueous solutions of PAA 100kDa 5 wt% at 21± 1 ^{0}C. A) Protonated PAA at pH=2, B) Ionized PAA at pH=13.

The degree of ionization, α, of PAA can be measured via FTIR spectroscopy of PAA solutions. The spectrum is reconstructed by linear superposition of the spectra obtained from hydrogenated PAA at pH=2 and ionized PAA at pH=13 in solutions of identical concentrations. The reconstruction of the spectrum was carried out by fitting α, according to equation 5 in the main text, and the method of least squares, and comparison of the obtained value to α calculated via potentiometric measurement.

In Figure S2 we present the measured (black curves) and fitted (red curves) spectra of PAA 100 kDa 5wt%.

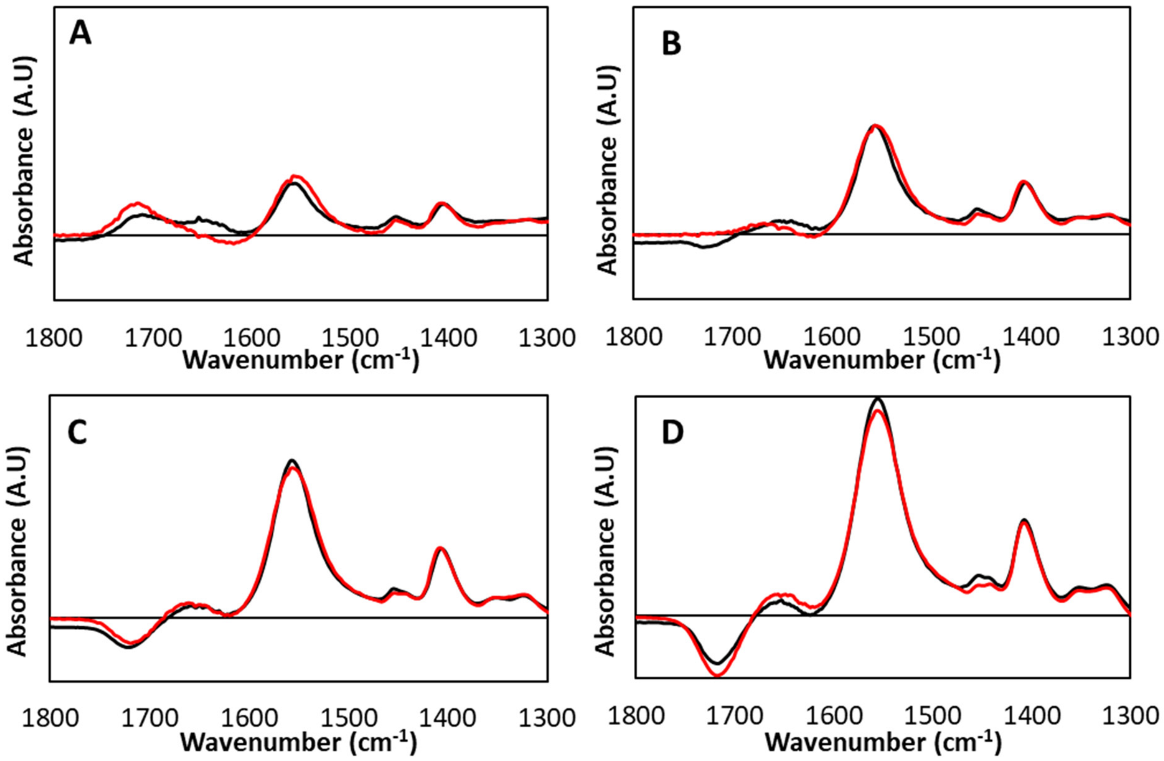

Figure S2. Re-constructed and measured spectra of PAA 30kDa 5wt% solutions. A) Degree of ionization, α = 0.3289 (fitted 0.3215), B) α = 0.5059 (0.5079), C) α = 0.6879 (0.6718), D) α = 0.8894 (0.8986) (ionized by NaOH solution 0.8951).

Figure S3. Degree of ionization as a function of pH in PAA solutions. A) PAA 100kDa 5wt% in water, B) PAA

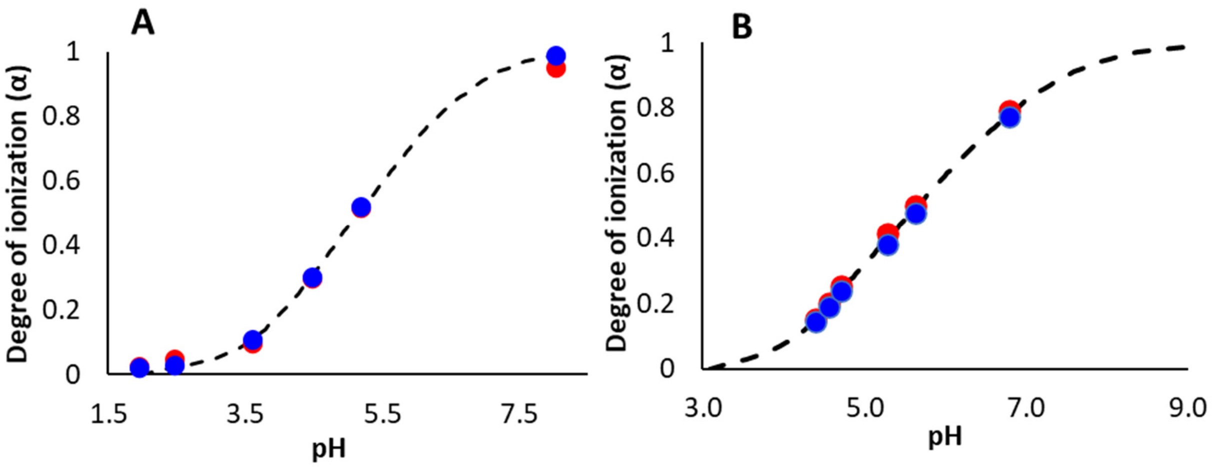

30kDa 1wt% + Brij-S20 1wt%. The dashed line is the calculated potentiometric titration curve. Blue dots (●) were obtained from potentiometry measurements, red dots (●) from FTIR.

Comparison of titration curves obtained via potentiometric method and direct ATR-FTIR measurement is presented in Figure S3.

Overlap concentration, C*, calculated from viscosity measurements

The viscosity of PAA solutions was measured via capillary flow for PAA 30 kDa and 100 kDa. Four different concentrations were measured for each molecular weight: For 30kDa 0.002< C < 0.009 gr/ml, and for 100kDa 0.002< C< 0.014 mg/ml, at 21±1 ^{0}C.

Figure S4. Viscosity measurements of PAA. A) 30kDa, B) 100kDa.

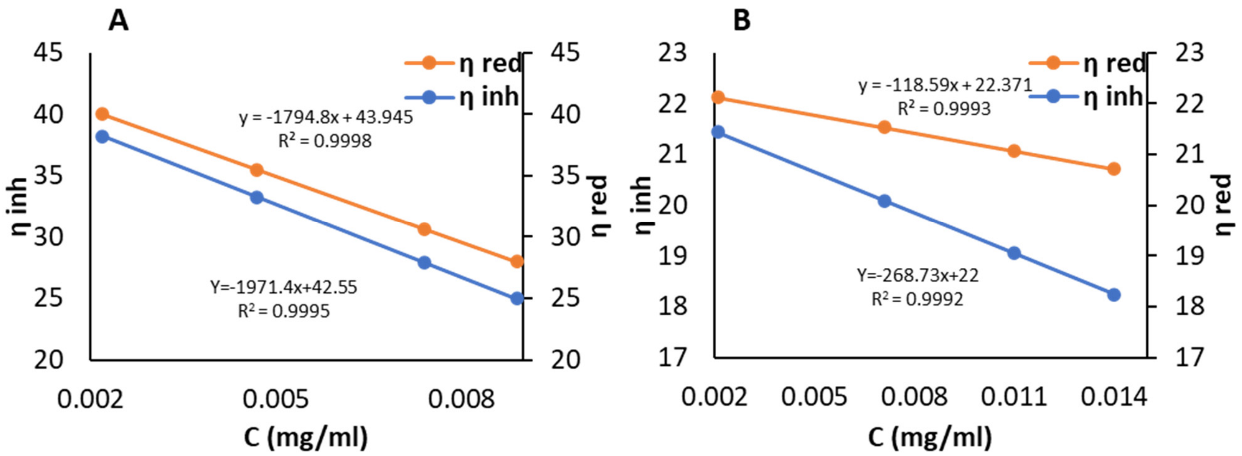

The inherent and reduced viscosities were calculated from equations (6) and (7). The value of C* was calculated by extrapolation of the reduced and inherent viscosity to C= 0 (mg/ml), C* (30kDa)= 2.31±0.05 wt%, and C* (100kDa)= 4.51±0.05 wt%.

The intrinsic viscosity of PAA 30kDa and 100kDa are 42.3±0.7 dL/gr and 22.2±0.2 dL/gr, respectively.

Titrations in micellar solutions

Titration experiments in micellar solutions were carried out. TEM imaging, of vitrified Brij-S20 solutions and dried, negatively stained F127 and F108 solutions was performed. Cryo-TEM images of solutions of Brij-S20 micelles in solutions of PAA 30 kDa at pH = 3.5 and pH = 7 are presented in Figure S5 A, B. The nanometric Brij-S20 micelles appear as dark dots on the background of the vitrified solution, showing the high density of micelles in the solutions. The solvated PAA cannot be observed in cryo-TEM. The typical diameter of Brij-S20 micelles of about 5-8 nm, as obtained from TEM measurements (averaged over 50 particles), is in agreement with previous reports [2].

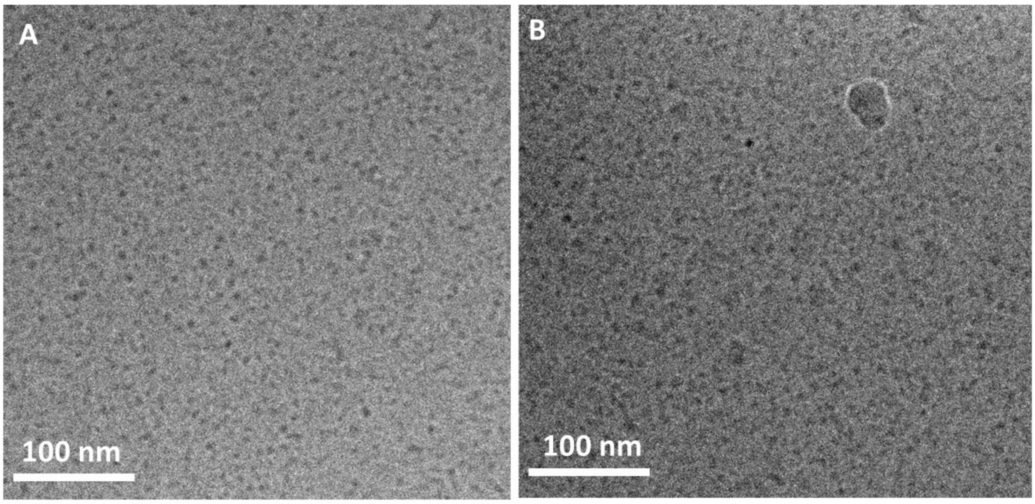

Figure S5. Cryo-TEM images of micellar solutions of A) Brij-S20 (1 wt%) in PAA 30kDa (1 wt%) pH= 3.5, and B) Brij-S20 (1 wt%) in PAA 30kDa (1 wt%) pH = 7.

TEM images of F108 solutions obtained by drying the samples and staining them with solution of Uranyl acetate are shown in Figures S6 and S7. The dark background results from the presence of the heavy Uranyl atoms. The bright spherical-like objects observed in Figure S6 B are attributed to the PPO core of the F127 micelles (diameter of 5-6 nm), while the highly hydrated and much less densely packed PEO shell (where the uranyl ions are not excluded) are not observed[3]. Note that 4 wt% F108 solutions are below the CMC of the polymer at 22 °C, and indeed micelles are

not observed in the dried, stained sample as presented in Figure S6 A. However, for 5 wt% F108 solutions, bright spherical-like objects are observed as in the image presented in Figure S6 B.

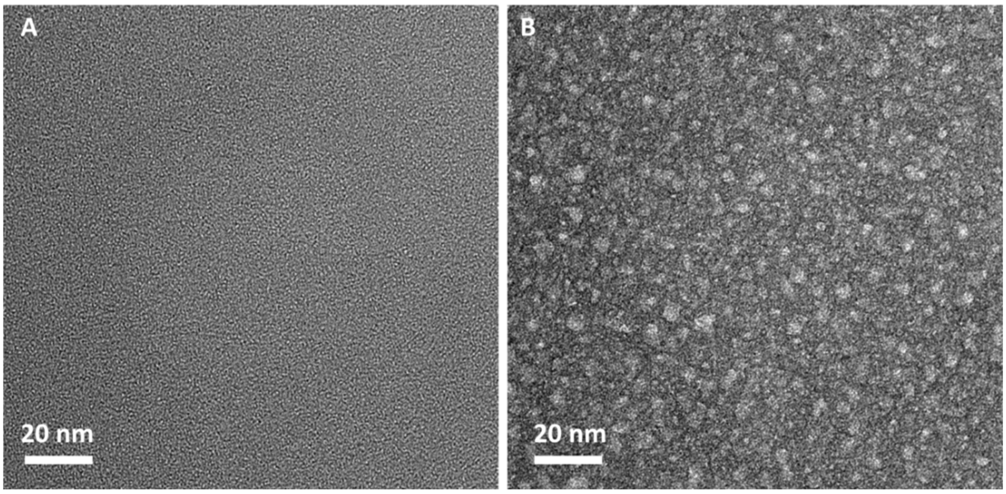

Figure S6. TEM images of negatively stained samples prepared from micellar solutions of F108 at pH = 7. A) 4wt% and B) 5wt%.

Figure S7 presents images of dried, negatively stained samples prepared from mixtures of F108 and PAA, at pH = 3.5 (Figure S7 A) and pH = 7 (Figure S7 B). The image is similar to that obtained from the native solution (Figure S6 B).

Figure S7. TEM images of negatively stained samples prepared from micellar solutions of F108 (5 wt%) in PAA

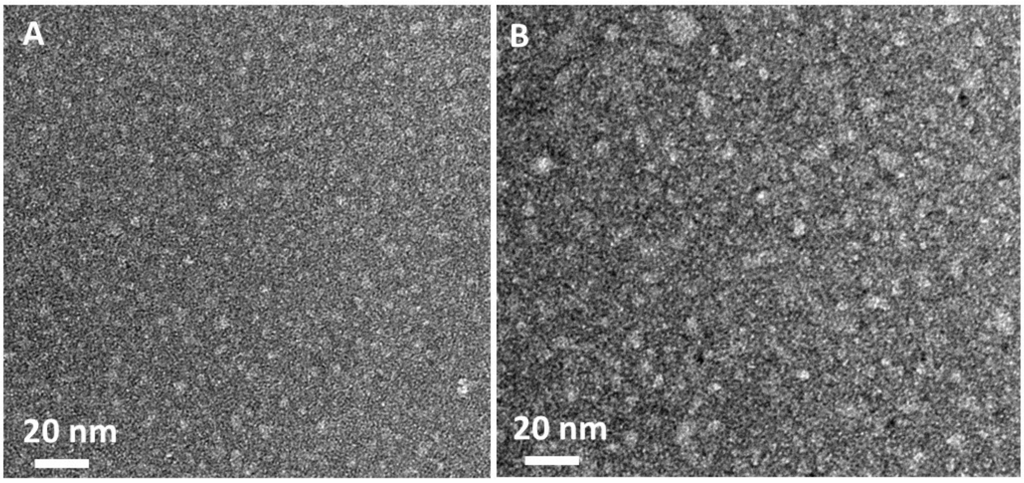

30kDa (1 wt%). A) pH= 3.5 and B) pH = 7.

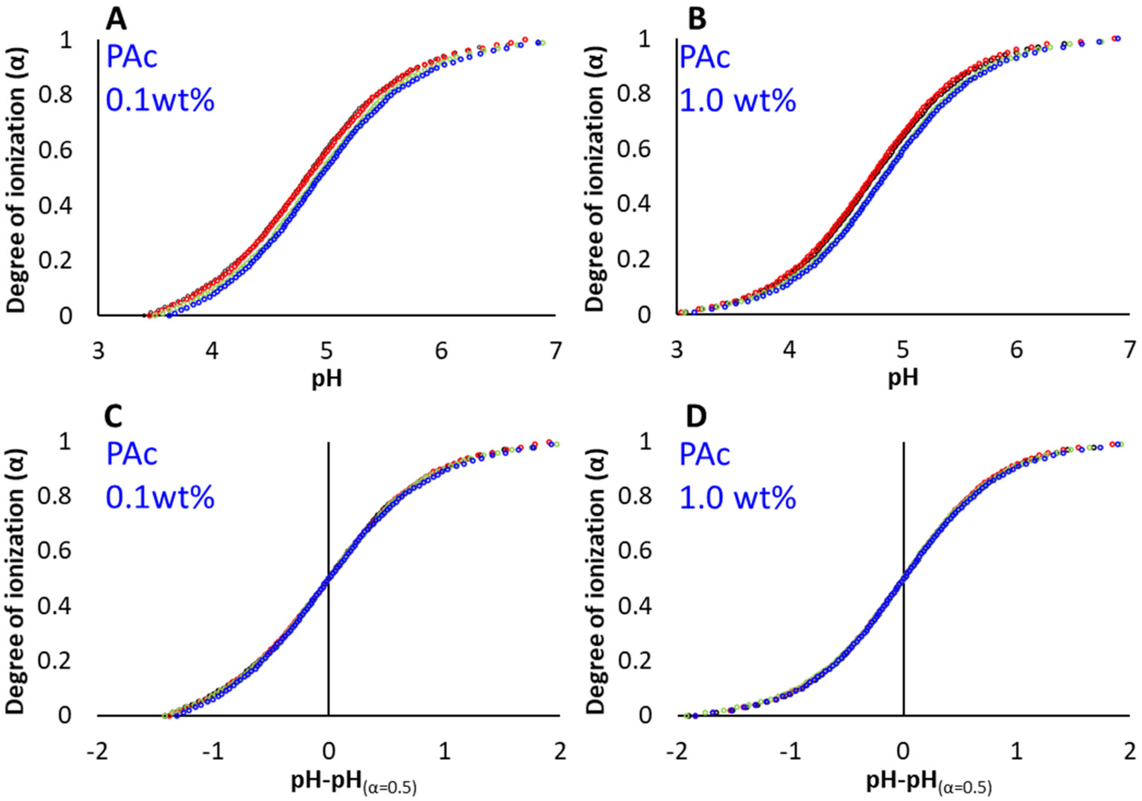

Figure S8. Degree of ionization as a function of pH of A) PAc 0.1wt%, B) PAc 1 wt%, and as a function of ΔpH (pH-pH$_{(\alpha=0.5)}$) C) PAc 0.1wt%, D) PAc 1wt%. In water (black), Brij-S20 1 wt% (red), F108 5 wt% (green), and F127 5wt% (blue).

Titrations of PAc in micellar solutions (Figure S8 A and B) show a constant shift of up to 0.1 pH unit as compared to that in micelle-free aqueous solutions of PAc (black curve), and the shape of the titration curve (Figure S8 C and D) is not modified.

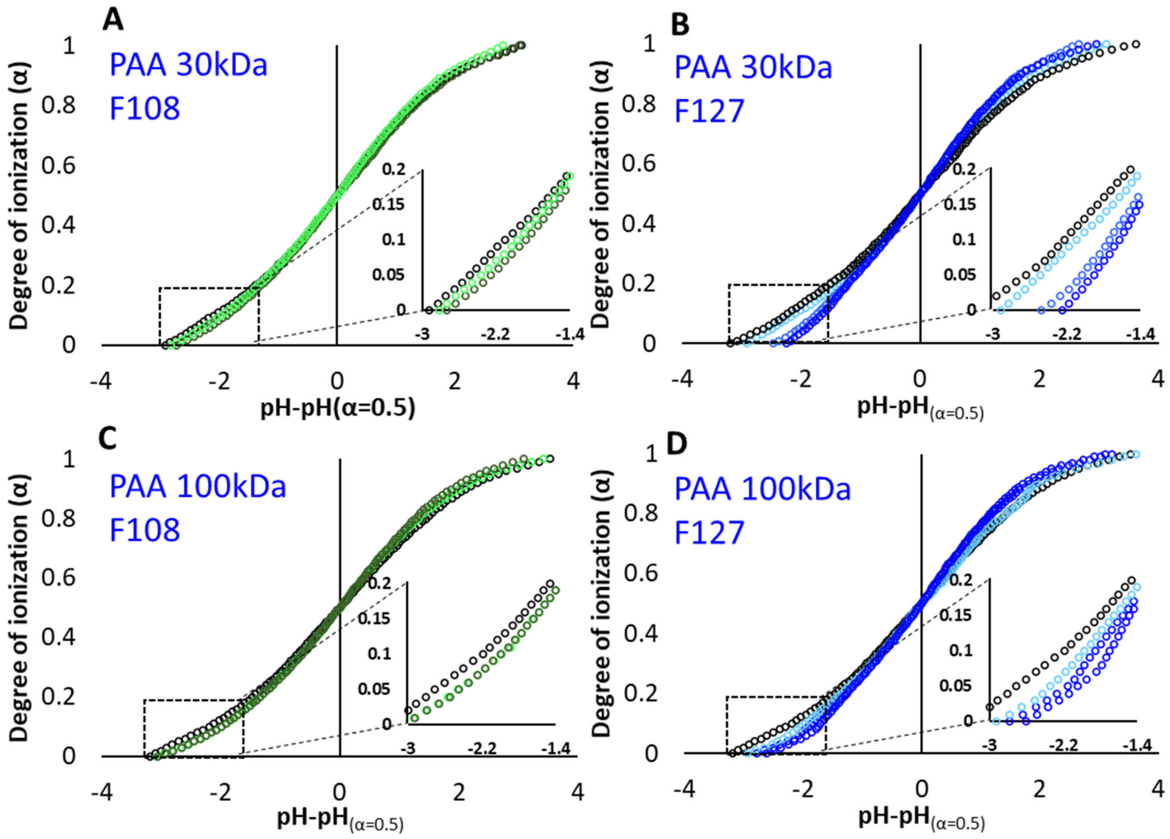

Figure S9. Titration curves of PAA (0.1 wt%) solutions containing different concentrations of F108 and F127. PAA 30 kDa (A and B), PAA 100 kDa (C and D). PAA in water (**black**), PAc in Pluronic solutions (5wt%) (dashed lines in A and C). A) PAA 30kDa in F108 3wt% (below the CMC, bright green) and in F108 5wt% (above the CMC, **olive green**). B) PAA 30kDa in solutions of F127 3wt% (**cyan**), 5wt% (**azure**) and 7%(**blue**). The same color code applies for PAA 100kDa presented in Figure 3 C, D.

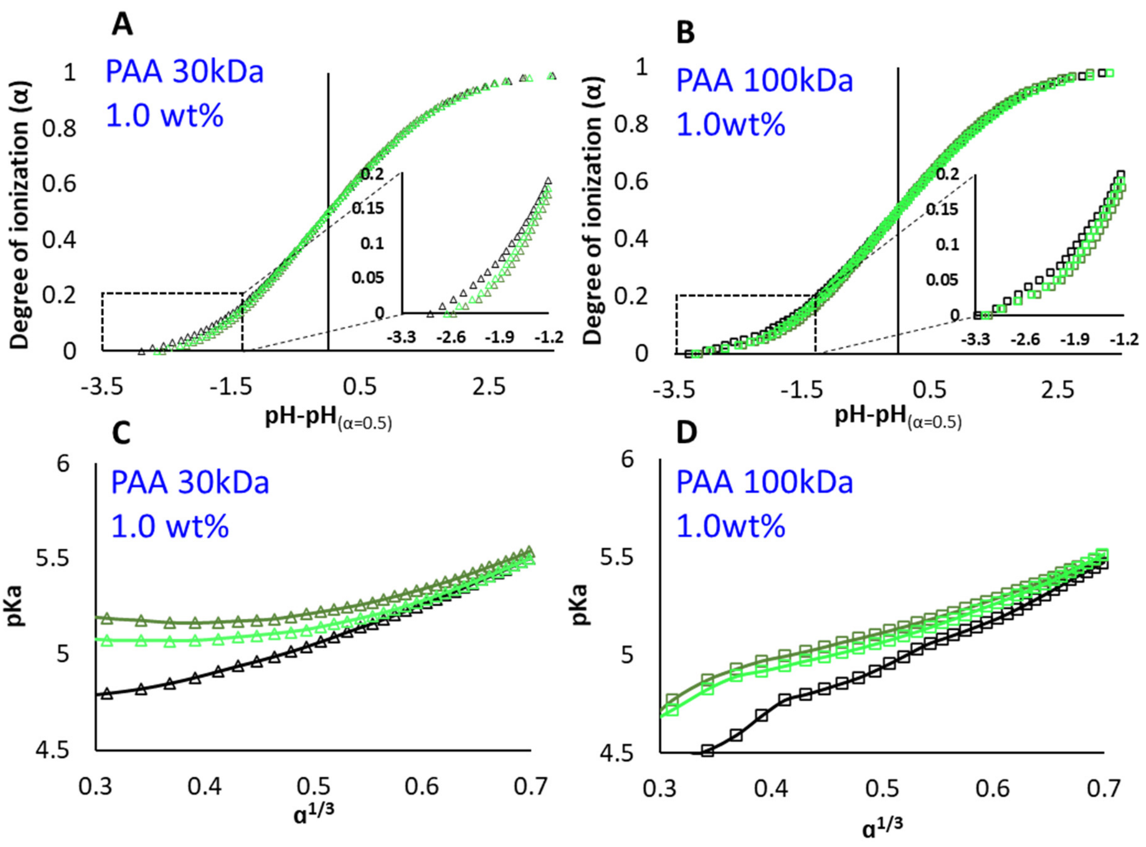

Figure S10. Titration curves of PAA in F108 solutions, below and above the CMC. A) 1 wt% PAA 30 kDa B) 1 wt% PAA 100 kDa. C, D) pKa as a function of $\alpha^{1/3}$ (equation 4). PAA in water (**black**), in solutions of F108 3wt% (below the CMC, **olive green**) and in F108 5wt% (above the CMC, **bright green**). C) 1 wt% PAA 30 kDa, D) 1 wt% PAA 100 kDa.

Surface tension measurements of F108 solutions

Surface tension measurements of mixtures of PAA (30 kDa, concentration of 0.1 wt%) and F108 are presented in Figure S11. As reported before[4] due to the high polydispersity of the polymer the CMC is not easily detected in the native solutions. While the absolute value of the curves is reduced due to the surface activity of PAA , the overall behaviour of the curves is not significantly modified by the presence of PAA in either pH. The calculated surface access, presented in Table S1, is similar in the PAA-free and PAA -F108 mixtures.

The surface access calculated via Gibbs adsorption isotherm[5]:

$$(S1)\ \Gamma = -\frac{1}{RT}\left(\frac{d\gamma}{d\ \ln C}\right)$$

Where R – gas constant, T – the absolute temperature, g – surface tension, C – molar concentration.

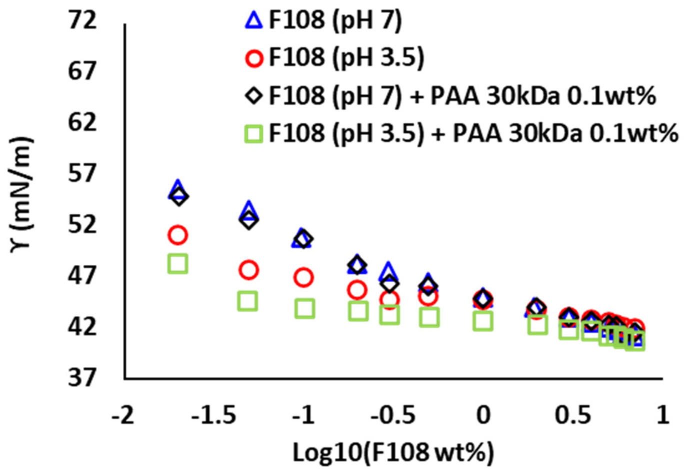

Figure S11. Surface tension vs. log F108 concentration at pH= 7 (blue) and pH=3.5 (red), and in solutions of PAA 30 kDa (0.1wt%) at pH =7 (black) and pH= 3.5 (green). Typical error of ± 0.1 mN/m.

Table S1. Surface access of surfactant molecule.

Micelle's type	Acid	pH	Surface access (10^{-3} mol/m^2)
Brij-S20	-	3.5	2.2
Brij-S20	-	7	2.4
Brij-S20	PAA 30kDa 0.1wt%	3.5	2.7
Brij-S20	PAA 30kDa 0.1wt%	7	3.7
F108	-	3.5	0.65
F108	-	7	1.0
F108	PAA 30kDa 0.1wt%	3.5	0.54
F108	PAA 30kDa 0.1wt%	7	0.95
F127	-	3.5	1.7
F127	-	7	1.6
F127	PAA 30kDa 0.1wt%	3.5	2.5
F127	PAA 30kDa 0.1wt%	7	1.5
F127	PAc 0.1wt%	3.5	1.2
F127	PAc 0.1wt%	7	1.5

Small -angle X-ray scattering characterization

As described in the experimental part of the main text, the scattering patterns obtained from PAA (30 kDa), F127, and Brij-S20 solutions were fitted to appropriate models using SASview [6]. These models are described below.

Polyacrylic acid (PAA): The scattering patterns of 1 wt% PAA (30 kDa) at pH = 3.5 and 7 are presented in Figure S12. At pH = 3.5, where the polyelectrolyte is expected to be uncharged, the

SAXS curve exhibits a typical scattering pattern of polymer in a solution; while at pH = 7 (> pKa of PAA), a correlation peak and a slight upturn characteristic to polyelectrolytes (and WPE in general) in salt-free solutions, below C*[7] are observed.

Therefore, the scattering curve of PAA at pH = 3.5, was fitted to the Debye Gaussian coil model[8]:

$$\text{(S2)} \qquad I(q) = 2 \cdot I_0 \cdot \frac{\left\{\exp\left[-\left(qR_g\right)^2\right] + \left(qR_g\right)^2 - 1\right\}}{\left(qR_g\right)^4}$$

Where I_0 is a scale factor, and R_g is the radius gyration of the polymer coil.

And the scattering curve of PAA at pH = 7 was fitted to the broad Lorentzian peak empirical model[9]:

$$\text{(S3)} \qquad I(q) = \frac{A}{q^n} + \frac{C}{1 + (|q - q_0|\xi)^m}$$

Where *A* is the Porod law scale factor and *n* is the Porod exponent, *C* is the Lorentzian scale factor, ζ is the screening correlation length (described in the main text), and *m* is roughly equal to $1/\nu$ where ν is the Flory exponent, which indicates the behavior of a polymer in a solvent (3/5 for good solvent, 1/2 for theta solvent)[10]. The peak maximum, q_0, corresponds to $d_0 = 2\pi/q_0$, representing an average distance between the charged PAA segments.

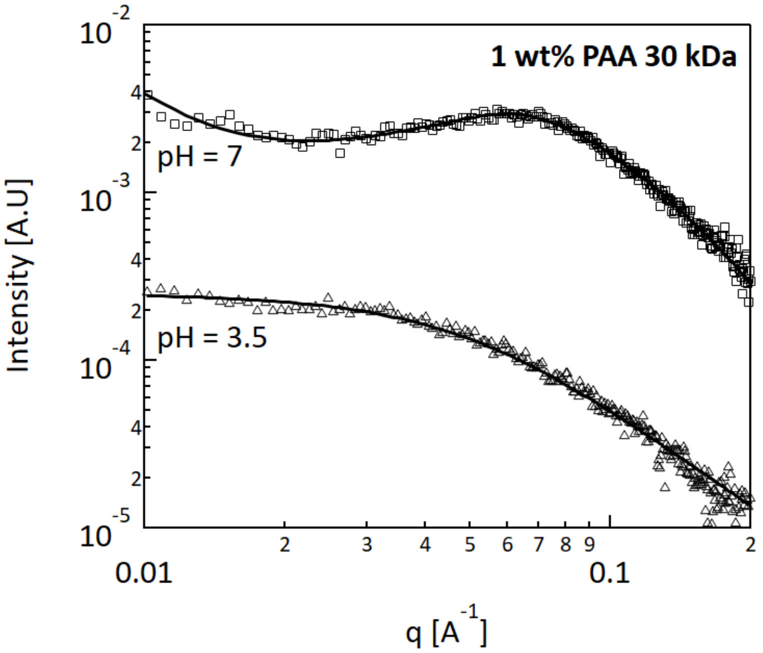

Figure S12. The scattering patterns of 1 wt% PAA (30 kDa) at pH = 3.5, and pH = 7. The solid black lines represent the best model fittings: the Debye Gaussian coil and broad Lorentzian peak models, respectively.

The best fit to equations S2 and S3 is presented as a solid black line (Figure S12), and the best-fit parameters are summarized in Table S2. The fitted parameters are in agreement with previous reports[11,12].

Table S2. Best fit parameters to Debye Gaussian coil[8] and broad Lorentzian peak empirical model[9], for PAA at pH = 3.5 and 7, respectively.

PAA pH = 3.5		PAA pH = 7						
I_0	R_g (nm)	A	n	C	q_0 (A^{-1})	d_0 (nm)	z (nm)	m
56	3.0 ± 0.1	$3.5 \cdot 10^{-9}$	2.7 ± 0.2	0.001	0.0602 ± 0.004	10.4 ± 0.1	1.9 ± 0.1	1.69 ± 0.05

* The obtained values of I_0 (corresponds to the Debye Gaussian coil model), A, and C (corresponds to the broad peak model) do not have a physical meaning since the measurements were not carried out in absolute units.

Pluronic-F127: The scattering profiles of F127 in water and F127 in PAc and PAA solutions were described by a core-shell sphere form factor combined with a hard-sphere structure factor. Polydispersity was applied to the sphere's core.

The core-shell-sphere form factor is given by:

$$(S4) \qquad P(q) = \left[\frac{4\pi R_c^3}{3}(\rho_c - \rho_s)\phi(qR_c) + \frac{4\pi R_s^3}{3}(\rho_s - \rho_{solv})\phi(qR_s)\right]^2$$

Where R_c, ρ_c, and R_s, ρ_s are the radii and the electron density of the core and shell, respectively. The normalized amplitude scattered by a sphere is $\phi(qR) = 3\frac{\sin(qR) - qR\cos(qR)}{(qR)^3}$.

The Schulz distribution represents the polydispersity of the core:

$$(S5) \qquad f(R) = \frac{K^{-1} \cdot (\beta)^\beta}{R_c\Gamma(\beta)} \cdot \exp\left[-\frac{\beta R}{R_c}\right] \cdot \left(\frac{R}{R_c}\right)^{\beta - 1}$$

Where K^{-1} is a normalization factor and β is the distribution width associated with the polydispersity of the micelle core δ_c by $\beta = \delta_c^{-2}$.

The hard sphere structure factor with Percus-Yevick closure[13] is described by the volume fraction ϕ and the hard-sphere interaction distance R_{HS}.

The best fits to the combined model are presented as a solid black line in Figure 5 of the main text, and the best-fit parameters are summarized in Table S3. The fitted parameters follow previous

reports[14]. Note that the scattering curve of F127-PAA at pH = 7 was not fitted to a model since the curve is a liner combination of the individual components.

Table S3. Best fit-parameters from core-shell-spheres model of the scattering curves of 5 wt% F127 in water, 1 wt% Pac, and 1 wt% PAA.

pH	Solvent	R_c [nm]*	δ	R_S [nm]	ϕ [v/v]	R_{HS} [nm]
3.5	Water	3.4	0.17 ± 0.02	7.8 ± 0.1	0.084 ± 0.005	11.2 ± 0.1
	1 wt% PAc	3.4	0.25 ± 0.02	7.4 ± 0.1	0.135 ± 0.004	10.8 ± 0.1
	1 wt% PAA	3.4	0.19 ± 0.01	5.8 ± 0.1	0.186 ± 0.001	9.2 ± 0.1
7	Water	3.4	0.27 ± 0.06	8.1 ± 0.7	0.087 ± 0.006	11.1 ± 0.1
	1 wt% PAc	3.4	0.36 ± 0.06	7.8 ± 0.1	0.177 ± 0.004	11.2 ± 0.1

* The value of R_c were fixed to 3.4 nm, as previously reported[14].

BrijS-20: Figure S13 presents the SAXS curves obtained from micellar solutions of Brij-S20 in water and in solutions of PAA and PAc, at pH = 3.5 (Figure S13A) and pH = 7 (Figure S13B). The scattering curve of Brij-S20 in water can be fitted to a monodisperse core-shell-sphere form factor (Equation S4). The scattering profile of Brij-S20 in 1 wt% PAA (30 kDa) is well described by the polydisperse core-shell-sphere model combined with hard-sphere structure factor (Equations S4, S5). The best fits to these models are presented as solid black lines and the best-fit parameters are summarized in Table S4. The fitted parameters follow previous reports[2]. The SAXS curves exhibit a similar trend as described in the main text for F127 (Figure 5), though less distinct. The scattering curves of Brij-S20 micelles are only slightly altered by the change in the pH, or by the presence of 1 wt% PAc (curve 2 in both pH = 3.5 (Figure 13A) and pH = 7 (Figure 13B). Differently, the scattering curves of Brij-S20 in the presence of 1 wt% PAA depend on the pH of the solution: while at pH = 7 (Figure S13 D) the measured scattering curve from Brij-S20-PAA mixture can be reconstructed by superposition of the scattering curves of the different entities, at pH = 3.5 (Figure S13 C) such superposition fails to reconstruct the measured curve. The significance of this observation is discussed in the Discussion section of the manuscript.

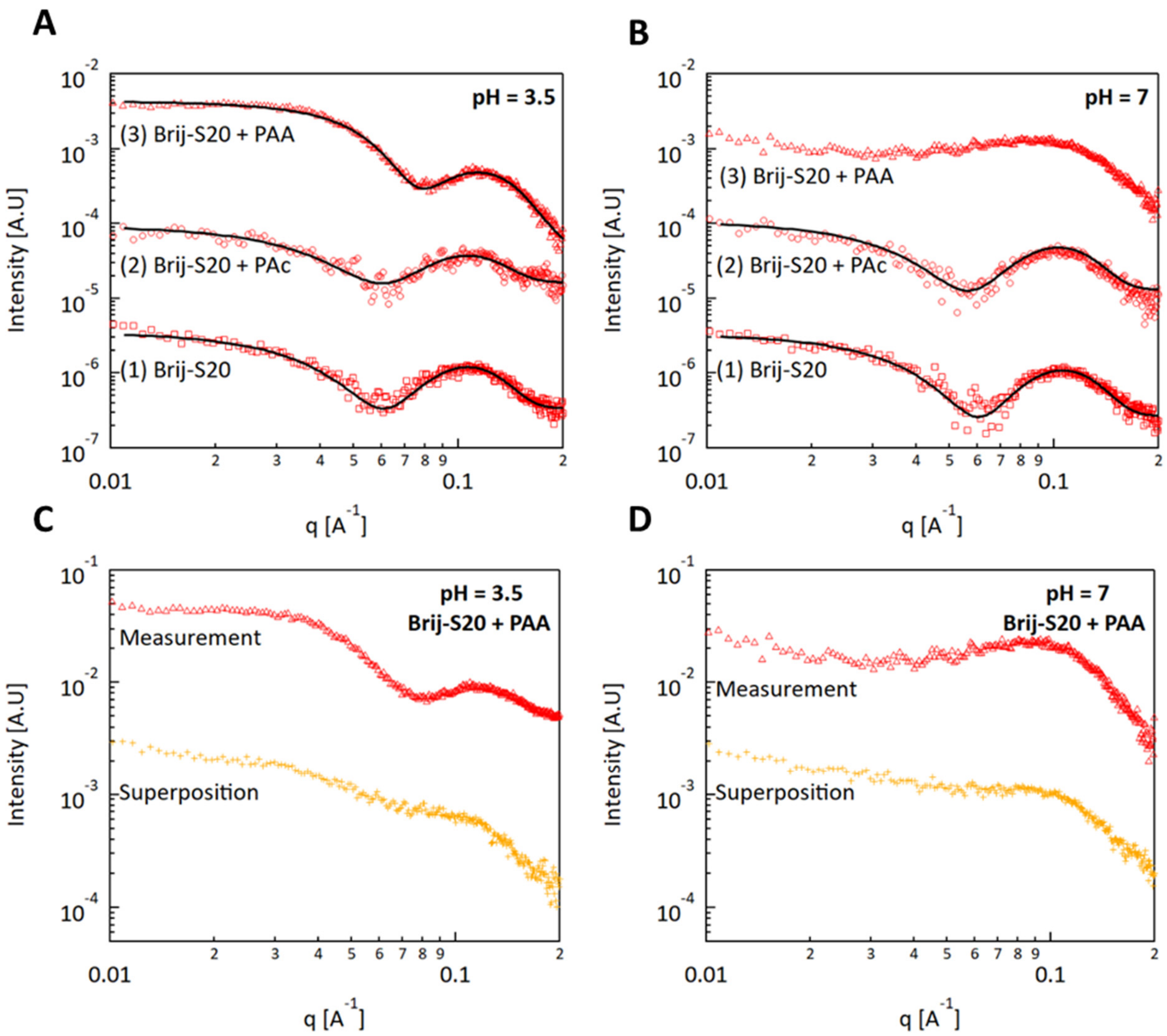

Figure S13. The scattering patterns obtained from mixtures of 1 wt% Brij-S20 in 1 wt% PAA (△), 1 wt% propionic acid (○), and 1 wt% Brij-S20 solution (□) at A) pH= 3.5 and B) pH = 7. Measured (△) and calculated via superposition (+) 1D SAXS curves obtained from 1 wt% Brij-S20 in 1 wt% PAA at C) pH = 3.5 and D) pH = 7. The solid black lines represent the best fit to equations S4-S5. The curves are shifted for better visualization.

Table S4. Best fit-parameters from core-shell spheres model of the scattering curves of 1 wt% Brij-S20 in water, 1 wt% PAc, and 1 wt% PAA.

pH	Solvent	R_c [nm]	δ	R_s [nm]	ϕ [v/v]	R_{HS} [nm]

		± 0.1		± 0.1		
3.5	Water	1.8		3.4	-	-
	1 wt% PAc	1.7	-	3.1	-	-
	1 wt% PAA	1.3	0.17 ± 0.02	3.2	0.0658 ± 0.006	4.6 ± 0.1
7	Water	1.9	-	3.2	-	-
	1 wt% PAc	1.9	-	3.3	-	-

Pluronics-F108: The nanostructure of 5 and 3 wt% F108 in water (CMC ~ 4.5 wt%, Table 2 of the main text) and F108 in the presence of PAc and PAA was investigated using SAXS (Figures S14, S15).

Figure S14A shows data obtained from solutions at pH = 3.5. Curve 1: 5 wt% F108 in water, F108+1 wt% PAc (curve 2) and F108 +1 wt% PAA (curve 3). The results exhibit a similar trend to that observed for F127 (Figure 5) and Brij-S20 (Figure S13), though less pronounced: while the presence of 1 wt% PAc has a minor effect on the scattering pattern of F108, PAA modifies the scattering curve of F108, due to crowding of the micelles. The scattering curve obtained from the mixture of F108 and PAA at pH = 7 (Figure 14B, curve 3) is a linear superposition of the PAA curve at that pH (Figure 5B (curve 3)) and can be reconstructed by linear superposition of the comprising curves (orange curve, Figure S14D). Very differently, at pH = 3.5 (Figure S14C) superposition of the curves does not reconstruct the measured curve from a mixture of PAA and F108.

Figure S15 presents the raw scattering data from solutions of F108 below the CMC (F108, 3 wt%) in water and PAA solutions at pH = 3.5 (Figure S15A) and pH = 7 (Figure S15B). At this concentration the relatively small core of PPO and the low contrast between PEO and water result in a curve that is not much different from that of water. In the presence of PAA (curve 3 in both A and B), the scattering is pH-depended, and while at pH = 7 (Figure 15B) it is a linear combination of the components, at pH = 3.5 (Figure 15A) the scattering curve of the mixture is significantly different.

We note here that while good fitting of the scattering pattern of F127 micellar solutions to a polydisperse core-shell-sphere form factor was obtained, the scattering curves obtained from F108 solutions and PAA-F108 could not be fitted to the model. Probably, due to the low contrast between the PEO blocks and water, and the relatively high polydispersity of the micelles (as can be seen in the dried-stained TEM images presented in Figure S7).

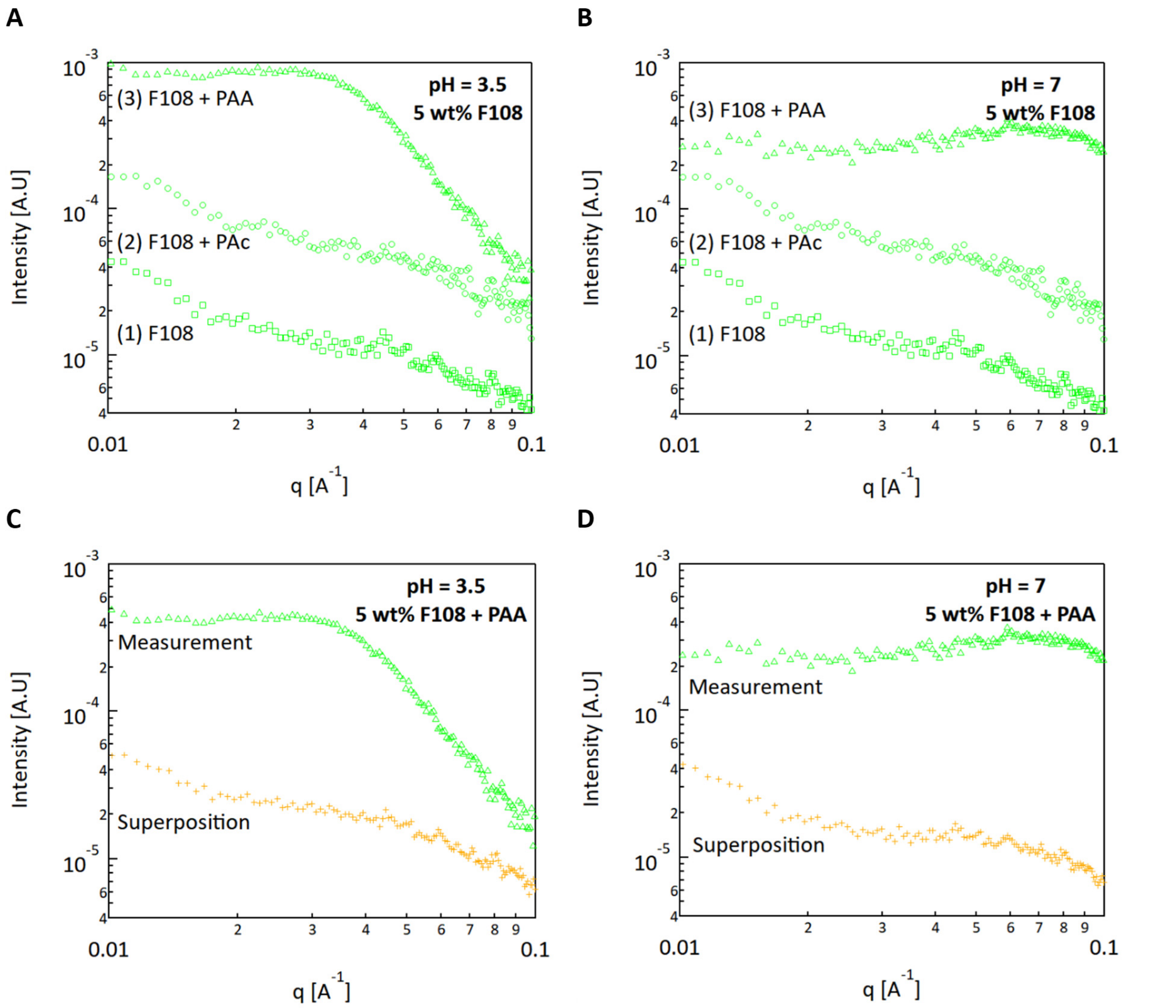

Figure S14. The scattering patterns obtained from mixtures of 5 wt% F108 in 1 wt% PAA (△), 1 wt% propionic acid (○), and 5 wt% F108 solution (□) at A) pH= 3.5 and B) pH = 7. Measured (△) and calculated via superposition (+) 1D SAXS curves obtained from 5 wt% F108 in 1 wt% PAA at C) pH = 3.5 and D) pH = 7. The curves are shifted for better visualization.

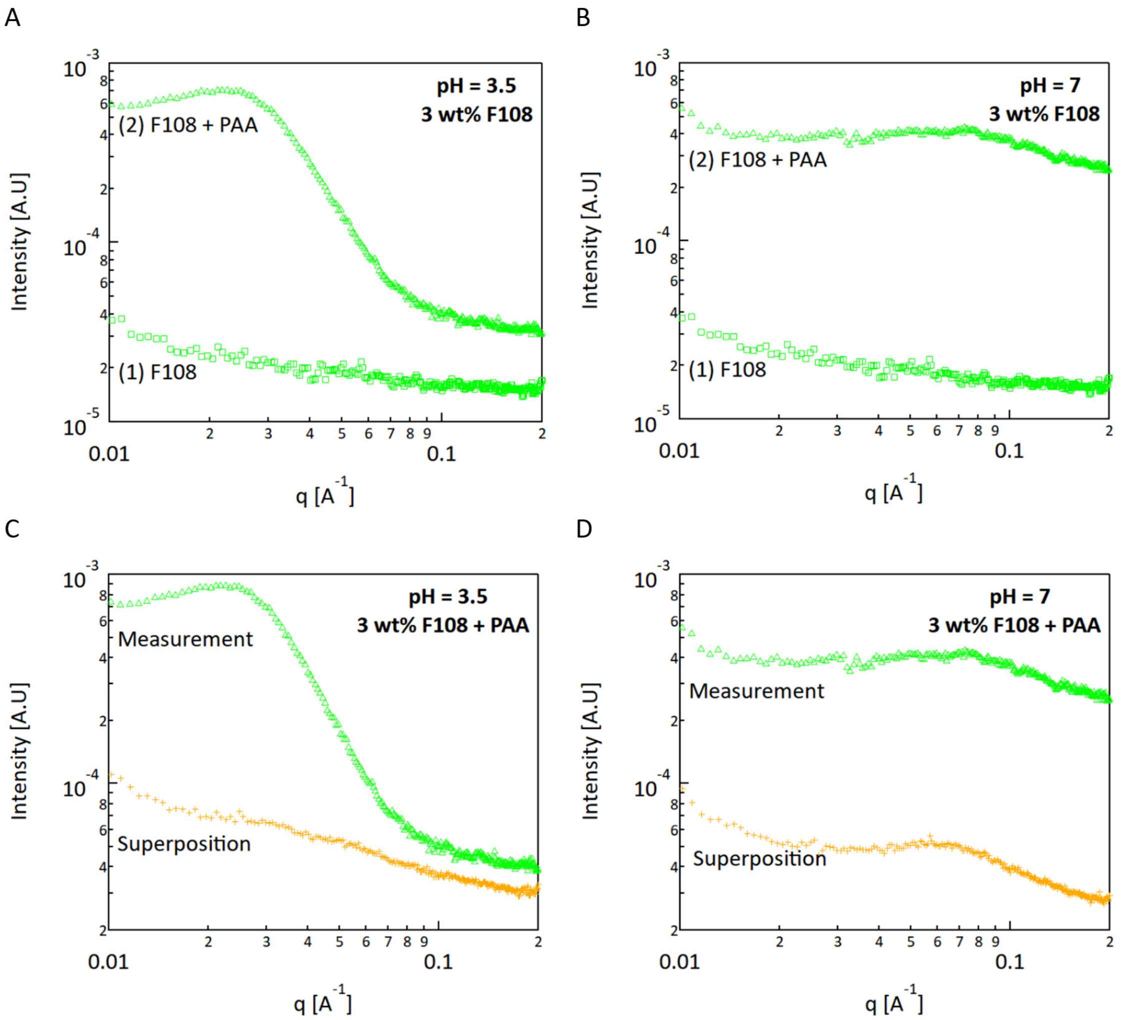

Figure S15. The scattering patterns obtained from mixtures of 3 wt% F108 in 1 wt% PAA (△), 1 wt% propionic acid (○), and 3 wt% F108 solution (□) at A) pH= 3.5 and B) pH = 7. Measured (△) and calculated via superposition (+) 1D SAXS curves obtained from 3 wt% F108 in 1 wt% PAA at C) pH = 3.5 and D) pH = 7. The curves are shifted for better visualization.

Figure S16 (A-C) summarizes the pKa shift of PAA (1 wt%) in micellar solutions of Brij-S20, F108 and F127. Figures S16 (D-G) presents the pKa as a function of $\alpha^{1/3}$ for PAA of 5kDa and 100kDa. Both show similar behavior to that of PAA 30kDa (Figure 3).

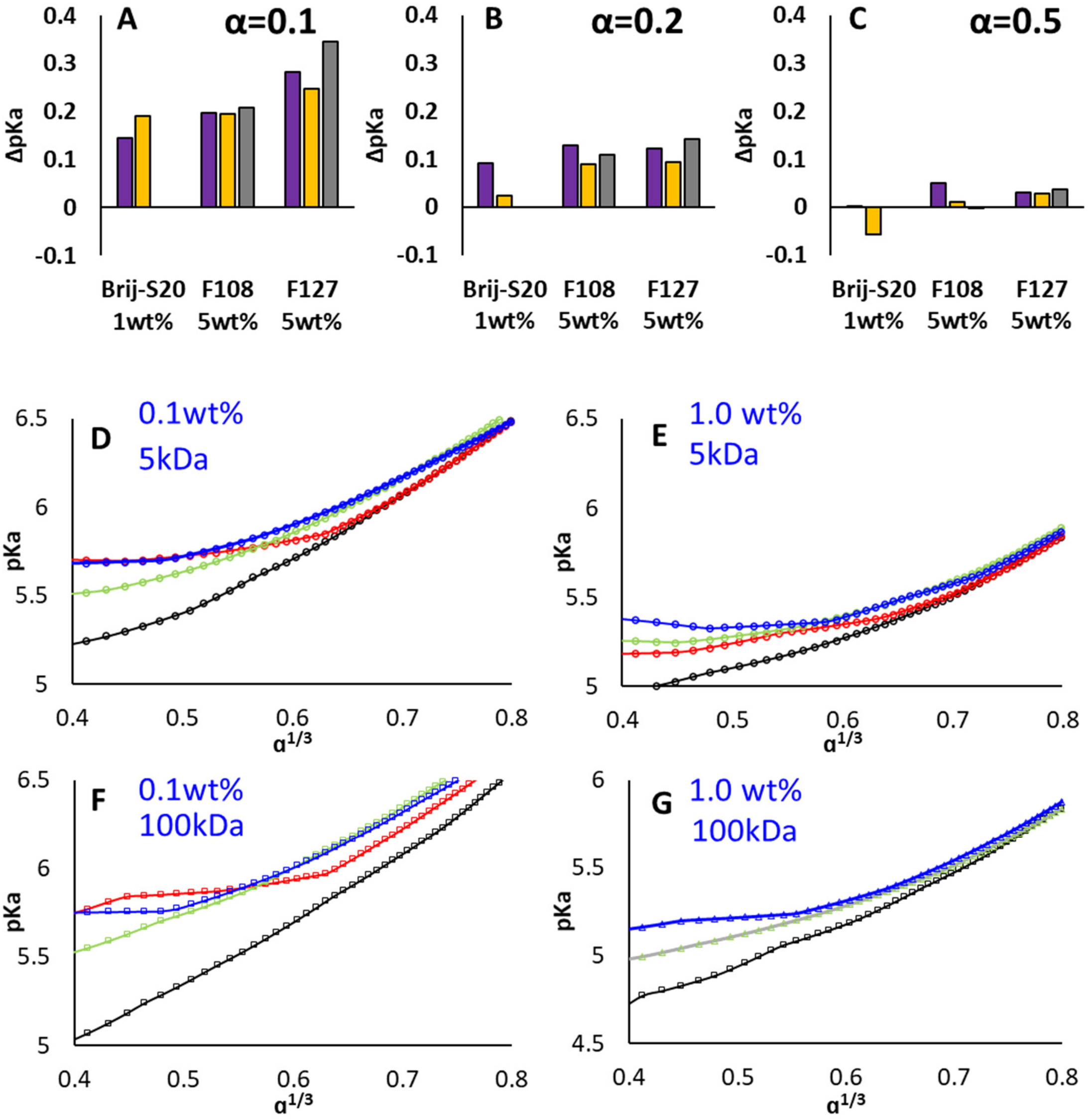

Figure S16. The deviation of pKa in micellar solutions (Brij-S20 1 wt%, F108 and F127 5 wt%) is presented for PAA (1 wt%): 5kDa (●, purple), 30kDa (●, yellow) and 100kDa (●, grey). A) α=0.1, B) α=0.2, C) α=0.5. pKa as a function of $\alpha^{1/3}$ (equation 4): D) PAA 5kDa (0.1wt%), E) PAA 5kDa (1 wt%), F) PAA 100kDa (0.1wt%), G) PAA 100kDa (1wt%). Micelles-free solutions (black), Brij-S20 1wt% (red), F108 5wt% (green), and F127 5wt% (blue).

References

1. Kirwan, L.J.; Fawell, P.D.; van Bronswijk, W. In situ FTIR-ATR examination of poly(acrylic acid) adsorbed onto hematite at low pH. *Langmuir* **2003**, *19*, 5802-5807, doi:10.1021/la027012d.
2. Ribeiro, M.; de Moura, C.L.; Vieira, M.G.S.; Gramosa, N.V.; Chaibundit, C.; de Mattos, M.C.; Attwood, D.; Yeates, S.G.; Nixon, S.K.; Ricardo, N. Solubilisation capacity of Brij surfactants. *International Journal of Pharmaceutics* **2012**, *436*, 631-635, doi:10.1016/j.ijpharm.2012.07.032.

3. Mortensen, K.; Talmon, Y. Cryo-TEM and SANS microstructural study of pluronic polymer solutions. *Macromolecules* **1995**, *28*, 8829-8834, doi:10.1021/ma00130a016.
4. Hadjichristidis, N.; Pispas, S.; Floudas, G. *Block copolymers: synthetic strategies, physical properties, and applications*; John Wiley & Sons: 2003.
5. Chattoraj, D. *Adsorption and the Gibbs surface excess*; Springer Science & Business Media: 2012.
6. Tan, L.X.; Elkins, J.G.; Davison, B.H.; Kelley, E.G.; Nickels, J. Implementation of a self-consistent slab model of bilayer structure in the SasView suite. *Journal of Applied Crystallography* **2021**, *54*, 363-370, doi:10.1107/s1600576720015526.
7. Taylor, T.J.; Stivala, S.S. Small-angle X-ray scattering study of a weak polyelectrolyte in water. *Journal of Polymer Science Part B-Polymer Physics* **2003**, *41*, 1263-1272, doi:10.1002/polb.10460.
8. Debye, P. Molecular-weight determination by light scattering. *The Journal of Physical Chemistry* **1947**, *51*, 18-32.
9. Horkay, F.; Hammouda, B. Small-angle neutron scattering from typical synthetic and biopolymer solutions. *Colloid and Polymer Science* **2008**, *286*, 611-620, doi:10.1007/s00396-008-1849-3.
10. Pedersen, J.S.; Schurtenberger, P. Scattering functions of semiflexible polymers with and without excluded volume effects. *Macromolecules* **1996**, *29*, 7602-7612, doi:10.1021/ma9607630.
11. Reith, D.; Muller, B.; Muller-Plathe, F.; Wiegand, S. How does the chain extension of poly (acrylic acid) scale in aqueous solution? A combined study with light scattering and computer simulation. *Journal of Chemical Physics* **2002**, *116*, 9100-9106, doi:10.1063/1.1471901.
12. Taylor, T.J.; Stivala, S.S. Small-angle X-ray scattering of poly(acrylic acid) in solution .1. Dioxane. *Polymer* **1996**, *37*, 715-719, doi:10.1016/0032-3861(96)87245-4.
13. Percus, J.K.; Yevick, G.J. Analysis of classical statistical mechanics by means of collective coordinates. *Physical Review* **1958**, *110*, 1.
14. Valero, M.; Hu, W.J.; Houston, J.E.; Dreiss, C.A. Solubilisation of salicylate in F127 micelles: Effect of pH and temperature on morphology and interactions with cyclodextrin. *Journal of Molecular Liquids* **2021**, *322*, doi:10.1016/j.molliq.2020.114892.
